# Effect of gamma irradiation on the wear behaviour of human tooth enamel

**DOI:** 10.1038/srep11568

**Published:** 2015-06-23

**Authors:** Ping Qing, Shengbin Huang, ShanShan Gao, LinMao Qian, HaiYang Yu

**Affiliations:** 1 State Key Laboratory of Oral Diseases, West China Hospital of Stomatology, Sichuan University, Chengdu, PR China; 2 Department of Prosthodontics, School and Hospital of Stomatology, Wenzhou Medical University, Wenzhou, PR China; 3 Tribology Research Institute, National Traction Power Laboratory, Southwest Jiaotong University, Chengdu, PR China

## Abstract

Radiotherapy is a frequently used treatment for oral cancer. Extensive research has been conducted to detect the mechanical properties of dental hard tissues after irradiation at the macroscale. However, little is known about the influence of irradiation on the tribological properties of enamel at the micro- or nanoscale. Therefore, this study aimed to investigate the effect of gamma irradiation on the wear behaviour of human tooth enamel in relation to prism orientation. Nanoscratch tests, surface profilometer and scanning electron microscope (SEM) analysis were used to evaluate the friction behaviour of enamel slabs before and after treatment with identical irradiation procedures. X-ray diffraction (XRD) and Fourier transform infrared spectroscopy (FTIR) were performed to analyse the changes in crystallography and chemical composition induced by irradiation. Surface microhardness (SMH) alteration was also evaluated. The results showed that irradiation resulted in different scratch morphologies, friction coefficients and remnant depth and width at different loads. An inferior nanoscratch resistance was observed independent of prism orientation. Moreover, the variation of wear behaviours was closely related to changes in the crystallography, chemical composition and SMH of the enamel. Together, these measures indicated that irradiation had a direct deleterious effect on the wear behaviour of human tooth enamel.

Radiotherapy, surgery, chemotherapy and a combination of these therapies constitute the basic treatments for oral-maxillofacial tumours, which are the most common cancer of the oral cavity worldwide^1^. However, even under the most careful protective planning, radiotherapy can damage the normal tissue and structures around the target^2^.

It is well-accepted that radiation-related dental caries are the most frequently observed irreversible type of damage^2^,^3^. Moreover, several studies have reported that the physical and chemical changes in teeth following irradiation contribute to the modification of mechanical properties, including the decrease of surface microhardness (SMH)^4^,^5^, ultimate tensile strength^6^ and fracture resistance^7^,^8^. Dental friction and wear, an inevitable process due to normal mastication, is of great importance in human daily life. Enamel, the hardest and most mineralized tissue in the human body, is exposed to the occlusal surface and chemical environment within the mouth. Thus, the anti-wear properties of enamel are critical for the health and normal function of human teeth.

Previous studies have shown that the tribological properties of enamel are significantly decreased when teeth are exposed to acidic environments (caries development or consumption of soft drinks)^9^,^10^ or tooth bleaching^11^. Excessive wear of teeth can result in disastrous consequences, including unacceptable damage to the occluding surfaces, alteration of the functional path of masticatory movement, dentin hypersensitivity, and even pulpal pathology. As mentioned above, several mechanical properties of enamel can be altered as a result of irradiation. However, no systematic study has analysed the effect of gamma irradiation therapy on the wear resistance of enamel. Moreover, the microstructural organization and relative composition of the organic, mineral, and water phases determine the mechanical properties of mineralized dental structures. However, little is known about the relationship between these factors and the tribological properties of enamel before and after irradiation. Therefore, we proposed, as the null hypothesis, that gamma irradiation has no effect on the tribological properties of enamel in terms of crystallinity, grain size and chemical component changes.

## Results

### Micro wear behaviours

**Figure 1:**
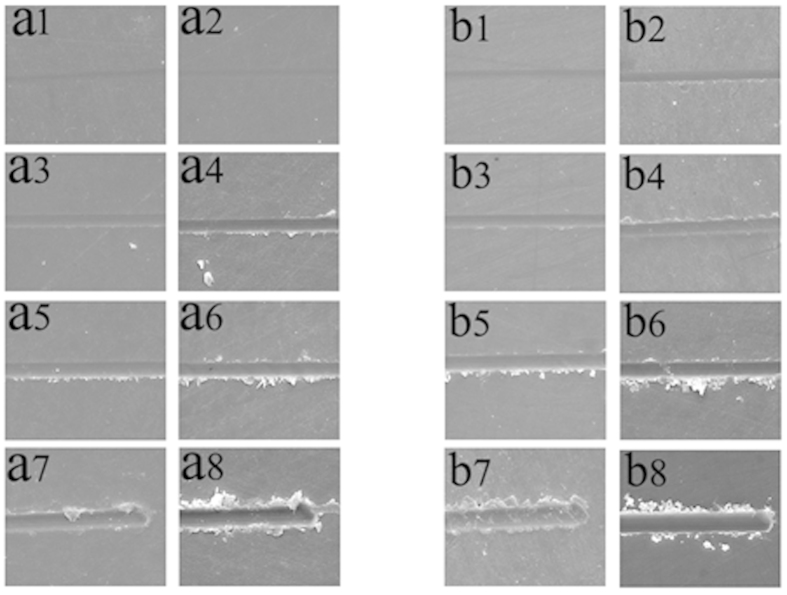
Tribological properties of enamel before and after irradiation. SEM images of scratches in the perpendicular-sectioned enamel slides before and after irradiation, subjected to different normal loads: (a1) Fn = 20 mN before irradiation; (a2) Fn = 20 mN after irradiation; (a3) Fn = 40 mN before irradiation; (a4) Fn = 40 mN after irradiation; (a5) Fn = 60 mN before irradiation; (a6) Fn = 60 mN after irradiation; (a7) Fn = 80 mN before irradiation; and (a8) Fn = 80 mN after irradiation. SEM images of parallel-sectioned enamel slides before and after irradiation, subjected to different normal loads: (b1) Fn = 20 mN before irradiation; (b2) Fn = 20 mN after irradiation; (b3) Fn = 40 mN before irradiation; (b4) Fn = 40 mN after irradiation; (b5) Fn = 60 mN before irradiation; (b6) Fn = 60 mN after irradiation; (b7) Fn = 80 mN before irradiation; and (b8) Fn = 80 mN after irradiation.

Figure 1a1-1a8 shows scanning electron microscopy (SEM) images of typical nanoscratch traces at different normal loads in perpendicular-sectioned enamel before and after irradiation. Distinct differences existed between the scratch morphologies of the enamel before and after irradiation. At a load of 20 mN, the grooves of the enamel both before (Fig. 1a1) and after irradiation (Fig. 1a2) were very shallow, and no obvious plastic deformation occurred. When the load was increased to 40 mN, a groove with clear edges became apparent due to significant plastic deformation. Meanwhile, no debris was found before irradiation (Fig. 1a3), while an obvious scratch with a small amount of debris at the edges of the scratch trace appeared after irradiation (Fig. 1a4). Under a load of 60 mN, a little debris accumulated at one edge of the scratch trace before irradiation (Fig. 1a5), whereas greater debris accumulated on edges along the length of the scratch traces after irradiation (Fig. 1a6). At the end of the scratch, more partial packing occurred with an increase in the load prior to irradiation (Fig. 1a7), and delaminations were observed at the edge of the scratch after irradiation (Fig. 1a8).

In the parallel to prism orientation, when the scratch ran parallel to the enamel rod, the scratch trace of the enamel before and after irradiation can be observed in Fig. 1b1b8. After irradiation, a small amount of debris appeared at one edge of the scratch trace under a load of 20 mN (Fig. 1b2), and the groove was clearer than the scratch trace of the enamel before irradiation (Fig. 1b1). With the load increased to 40 mN, a small amount of debris occurred around the scratch before irradiation (Fig. 1b3), whereas considerable debris was seen at two edges of the trace after irradiation (Fig. 1b4). Under a high load of 60 mN, more debris accumulated at the edge of the scratch trace before irradiation (Fig. 1b5), and a few delaminations with greater particle packing emerged after irradiation (Fig. 1b6). When the load was increased to 80 mN, additional debris formation was observed prior to irradiation (Fig. 1b7), and several obvious radial cracks nucleated and propagated outside the trace after irradiation (Fig. 1b8).

### Micro friction behaviours

**Figure 2:**
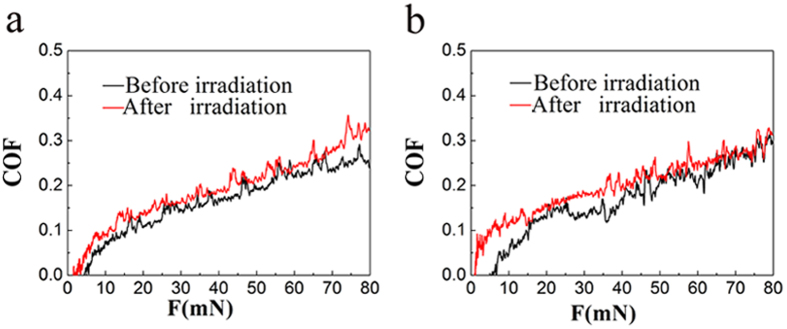
Friction coefficient-load curve of enamel before and after irradiation under a progressively increasing load in perpendicular-sectioned enamel slides (**a**) and parallel-sectioned enamel slides (**b**).

Figure 2a,b show the typical profile of the coefficient of friction (COF) in progressively increasing load mode for both perpendicular-sectioned and parallel-sectioned enamel before and after irradiation, respectively.

In the enamel with a perpendicular-to-prism orientation before irradiation (Fig. 2a), the friction coefficient could not be analysed at a load lower than 5 mN. However, as the load increased to 17 mN, obvious oscillation appeared. Then, with the normal load increasing further, the friction curve increased slowly and was accompanied by some fluctuations. The friction coefficient of specimens tested perpendicular to prism orientation after irradiation could not be obtained when the load was lower than 2 mN (Fig. 2a). When the load approached 13 mN, distinct oscillations appeared in the friction curve. Then, the friction coefficient increased slowly, along with the appearance of some fluctuations, as the normal load increased. In general, the enamel after irradiation exhibited a friction coefficient higher than that of the enamel before irradiation. Additionally, higher normal loads were associated with higher friction coefficients.

In the enamel with a parallel to prism orientation, Fig. 2b shows a curve of the friction coefficient of enamel samples before and after irradiation. The friction coefficient of enamel before irradiation could not be recorded when the load was 8 mN. However, as the load increased further, the coefficient of friction increased slowly and was accompanied by obvious fluctuations. In enamel after irradiation, the friction curve was similar to that in enamel before irradiation, apart from a higher friction coefficient compared to enamel before irradiation at the same load.

### Residual depth and residual width

In the enamel with a perpendicular to the prism orientation, typical scratch profiles at a constant load (20, 40 and 60 mN) are shown in Table 1. It should be noted that the scratch depth increased significantly in enamel after irradiation compared with enamel before irradiation at loads of 40 mN and 60 mN, although the depth did not achieve statistical significance when the load was 20 mN. Meanwhile, the scratch width increased with the load, but this difference was not statistically significant.

Table 1 also shows the variation in scratch depth at a constant load for enamel in the parallel to the prism orientation. The residual depth increased with the load; under a load of 40 mN and 60 mN, the scratch depths in enamel after irradiation were significantly deeper compared with the enamel before irradiation. This depth did not reach statistical significance when the load was 20 mN. The results also showed that the width increased with an increase in the load, but this difference was not statistically significant.

### XRD results

**Figure 3:**
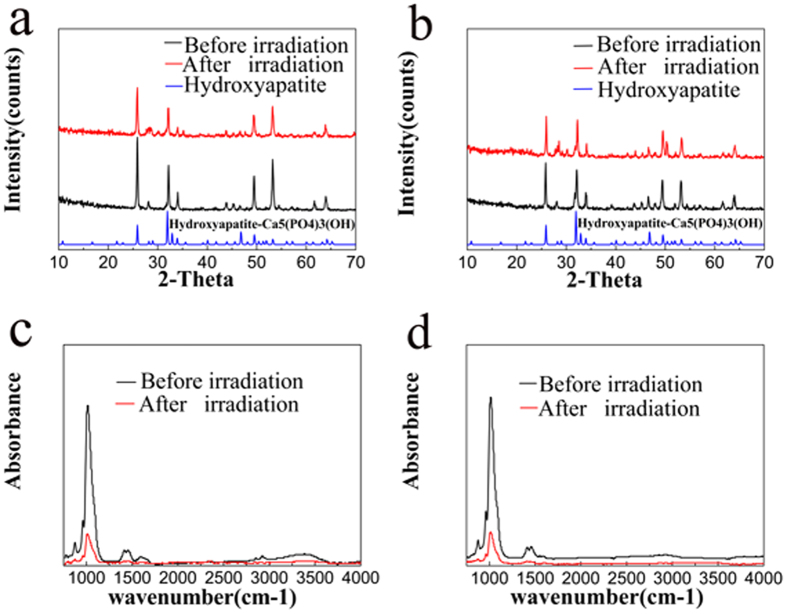
XRD patterns of enamel before and after irradiation. Perpendicular-sectioned enamel (**a**); Parallel-sectioned enamel (**b**). FT-IR spectrum of enamel before and after irradiation. Perpendicular-sectioned enamel (**c**); Parallel-sectioned enamel (**d**).

X-ray diffraction (XRD) spectra of the enamel before and after irradiation are shown in Fig. 3a,b. Experimental enamel XRD patterns were identified as hydroxyapatite, and the peaks were consistent with the standard hydroxyapatite diffraction pattern (JCPDS 86-0740). Meanwhile, the XRD analysis indicated that irradiation decreased the crystallinity of enamel and enlarged the crystal size both in perpendicular-sectioned enamel and parallel-sectioned enamel slides (Table 2).

### FTIR analysis

Based on the representative Fourier transform infrared spectroscopy (FTIR) spectra shown in Fig. 3c,d, both the absorbance and the integrated area of CO_3_^2−^ v2 and PO_4_^3−^ v1, v3 significantly decreased in the perpendicular-sectioned and parallel-sectioned enamel slides after irradiation. Furthermore, the PO_4_^3−^ v1, v3 integrated area decreased more than the CO_3_^2−^ v2 area. The results of the paired t-test revealed a significant increase in the carbonate: mineral ratio (C: M) after irradiation in both orientation groups (Table 2).

### Microhardness test results

Table 3 shows the results of SMH analyses of enamel in both the perpendicular- and parallel-to-prism orientation. There was no significant difference between enamel slabs before treatment. However, after irradiation, a statistically significant reduction in SMH was found in both perpendicular-sectioned enamel and parallel-sectioned enamel slides.

## Discussion

Radiotherapy is a basic treatment protocol that is widely used to manage head and oral cancer. As the tribological properties of enamel play an important role in the normal function of the tooth, an understanding of the wear behaviour of human irradiated enamel is of special interest in determining the quality of life of a patient with head and oral cancer. In the present study, we reported that commonly used gamma irradiation eventually reduced the wear resistance of enamel. This alternation may be related to changes in enamel crystallography and enamel chemical composition; therefore, the null hypothesis was rejected.

Recently, more research has focused on the tribological behaviours of materials at the microscale and nanoscale^12^,^13^,^14^ to characterize early damage to hard tissues and the alteration of microstructure caused by external factors. In the current study, the nanoscratch technique was applied to determine the microtribological characterization of enamel before and after irradiation. The results showed that normal enamel without exposure to irradiation in perpendicular-sectioned samples had a low friction coefficient, decreased debris formation and brittle delamination. However, when the scratch ran parallel to the enamel rods, more material removal and a higher friction coefficient were observed. All of these results are consistent with those of previous studies^9^,^14^. Interestingly, enamel in the perpendicular-sectioned and parallel-sectioned slabs after gamma irradiation showed a higher friction coefficient compared to enamel before irradiation. It is known that the wear resistance of enamel decreases with an increase in the friction coefficient^9^,^15^; therefore, the higher friction coefficient and deeper scratch remnant depth of enamel observed after irradiation (Fig. 2) indicated inferior anti-wear properties of the enamel. These observations might help explain the frequently observed partial to total enamel delamination after irradiation that is seen clinically^2^.

The microstructure of enamel has an effect on its mechanical properties and tribological behaviors^16^,^17^. Among different techniques, XRD was used to analyse the changes in the crystalline structure of human tooth enamel^18^. In our experiment, we found that irradiation induced a reduction in enamel crystallinity and enlarged crystals from XRD analysis (Table 2). The crystallinity of enamel has been widely reported to play an extremely important role in its mechanical properties^19^,^20^. Given that, the reduction in crystallinity indicated decreased mechanical properties, such as a reduction in hardness^21^. Sound enamel with a high crystallinity shows excellent mechanical and anti-wear properties. In our study, the enamel crystallinity significantly decreased in both perpendicular-sectioned and parallel-sectioned enamel segments after irradiation, which may account for the inferior nanoscratch resistance. On the other hand, it has been reported that enamel composed of larger crystals appears to be softer than enamel composed of small crystals^22^. Therefore, an age-related increase in enamel crystal size might be a reason for reduced tooth wear resistance in older individuals^23^. From the results of our study, enlarged crystals were observed in the enamel after irradiation, which might contribute to the reduction of nanoscratch resistance in enamel after irradiation.

Furthermore, previous studies have demonstrated that changes in enamel component alteration could influence nanoscratch resistance^16^,^17^. FTIR, an absorption spectroscopy technique that can examine inorganic materials and measure the quantitative alterations in the composition of mineralized tissue^24^, was applied in this study. The results showed that the integrated area of PO4^3−^ v1, v3 significantly decreased and the C: M ratio dramatically increased, which indicated that irradiation resulted in reduction of the mineral content of the enamel. On the one hand, from the results of the microhardness test, we clearly observed decreased SMH after irradiation. As noted by Panighi, there is a positive correlation between hardness and the mineral content of the tooth^25^. The reduction in mineral content in enamel after irradiation, as obtained by FTIR, was completely in agreement with the results of the microhardness test (Table 3). On the other hand, microhardness, a typical feature of the mechanical properties of enamel, can reflect the enamel’s susceptibility to abrasive wear^26^,^27^,^28^. Based on the results above, we may conclude that decreased microhardness and mineral content may lead to poor wear resistance of enamel after irradiation.

In 1975, Walker used an *in vitro* experiment to demonstrate the degradation of enamel proteins after irradiation, which destroyed the interaction of the organic matrix with apatite crystals^29^. Other researchers have suggested that a reduction in the mineral-organic interaction after irradiation would result in brittleness and an inferior fracture resistance of the enamel^5^,^30^. This alteration in the microstructure of enamel may also account for the inferior wear resistance in the present study. However, more experiments should be performed to demonstrate this phenomenon in future work.

Overall, our *in vitro* study indicated that the changes in crystallography and composition of enamel after irradiation may contribute to a reduction in the enamel’s wear resistance. To avoid this alteration in mechanical properties, continuous follow-up of the patients before, during, and after irradiation treatment should be provided. A recent study conducted by Soares *et al.* clarified that rinsing with 0.05% sodium fluoride would improve the mechanical properties of the irradiated enamel similarly to the non-irradiated enamel^31^. Therefore, understanding the wear resistance of the tooth after irradiation and the usage of substances to maintain mechanical properties during radiation treatment are important for reducing the side effects of radiotherapy and improving the quality of life of patients with cancer of the head and neck.

## Materials and methods

### Tooth collection and specimen preparation

All human teeth were collected from individuals aged between 13 and 20 years for orthodontic reasons after the patient signed an informed consent. The experimental procedures were approved by the Ethics Committee of the West China College of Stomatology, Sichuan University (WCHSIRB-ST-2013-152), and the methods were carried out in accordance with the Declaration of Helsinki (2008). All human teeth were collected and placed in normal saline (4°C) immediately after extraction.

**Figure 4:**
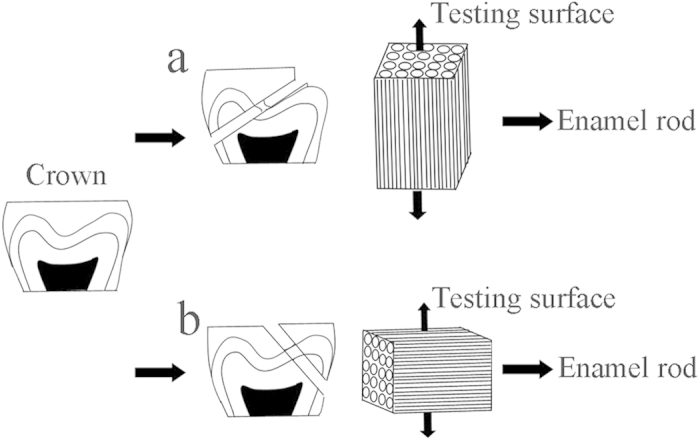
Specimen preparation. The cutting directions were adjusted to make the testing surface perpendicular (**a**) or parallel (**b**) to the direction of the enamel rod.

A total of nineteen human teeth were used in this study. Enamel slides were sectioned from each tooth using a diamond-coated band saw (Minitom; Struers, Copenhagen, Denmark) under running water. The cutting direction was adjusted to be perpendicular (2 slides) or parallel (2 slides) to the direction of the enamel rod (Fig. 4). Thirteen human teeth were randomly selected for scratch resistance and microhardness testing. These enamel blocks were embedded using polymethyl methacrylate, with a 2 mm × 2 mm exposure window for treatment. Another six human teeth, not imbedded in polymethyl methacrylate, were prepared for XRD and FTIR analysis. All of the specimens were first ground using silicon carbide papers (500, 800, 1,200, 2,000, 3,000, or 4,000 grit) in sequence and then polished with diamond paste of 10, 5, or 2.5 μm in turn. Grinding and polishing were conducted under water-cooling conditions in order to avoid the dehydration caused by local overheating. All of the specimens were then washed ultrasonically for 10 min with distilled water to remove debris, according to the method described in our previous studies^10^,^32^.

### Gamma irradiation procedure

Enamel specimens received 60 Gy of direct gamma radiation in a ^60^Co irradiation unit (GWXJ80 ^60^Co radiotherapy treatment unit, Nuclear Power Institute of China, China), with an exposure to daily increments of 2 Gy on 5 days a week for six weeks. This dose was defined based on the irradiation unit. The total dosage of radiation and the course of therapy were in accordance with those normally used for oral cancer patients to simulate the clinical situation^6^,^31^,^33^. All of the samples were immersed in beakers with artificial saliva changed daily, apart from irradiation and testing at room temperature. The artificial saliva was prepared according to a previous study, and the saliva consisted of 0.375 g/l CaCl2·2H2O, 0.125 g/l MgCl2·6H2O, 1.2 g/l KCl, 0.85 g/l NaCl, 2.5 g/l NaHPO4·12H2O, 1 g/l sorbine acid, 5 g/l carboxymethylcellulose sodium, and 43 g/l sorbitol solution (70%, noncrystalline)^34^. After irradiation, the specimens were thoroughly rinsed with deionized water and then tested.

### Nanoscratch tests

Nanoscratch tests were conducted with a CSEM nanoscratch tester apparatus (CSEM Instruments, Switzerland) under the same environmental conditions, using a conical diamond tip with a 2-μm radius. The enamel slabs before and after irradiation were tested according to prismatic orientation (transverse or parallel) without the application of artificial saliva at room temperature. The scratch test was performed with a progressive load from 0.1 to 80 mN with a velocity of 500 μm/min. The scratch distance was 500 μm. At least two scratches were made in each test region. Each scratch was at least 2 μm away from the subsequent scratch. Then, a series of 200-μm scratch tests were performed at constant normal loads of 20 mN, 40 mN and 60 mN. The residual depths of the scratch grooves under the constant load were measured with an Ambios XP-2 stylus profilometer (XP-2, Ambios Technology, Inc., USA). All morphologies were carefully observed using SEM (INSPECT F, Czech Republic) to reveal the deformation and fracture patterns, according to our previous study^26^.

### X-Ray diffraction

The crystal structure of the enamel specimens before and after irradiation was evaluated by X’pert XRD (X’pert PRO, Panalytical, Netherlands) with CuKα radiation at 35 kV/25 mA. Data were collected in the 2θ range of 10°–70°. Both the crystallinity and grain size were calculated using the software Jade 5 (MDI, Materials Data Inc., USA). The crystallite sizes were calculated using Scherrer’s formula^35^,^36^ as follows: D = 0.89 λ/β cosθ, where λ represents the wavelength (CuKα), β is the full width at the half-maximum of the HA (211) and θ is the diffraction angle.

### FTIR detection

FTIR spectrometric analysis was performed with a spectrometer (IRPRestige-21, Shimadzu, Japan). The spectra were recorded in the range of 650–4,000 cm^−1^ at a 4 cm^−1^ resolution. To maintain the measurement at the same place before and after irradiation, the reverse side of the testing surface was marked using a carbide bur before irradiation. The testing surfaces were then positioned against the diamond crystal of the FTIR unit and pressed with a force gauge at a constant pressure to facilitate contact. The spectra of the enamel before and after irradiation were obtained. Data were recorded and analysed with OMNIC 8.0 software (Nicolet, Madison, WI, USA). The band between 810 and 885 cm^−1^ represents CO_3_^2−^ v2, while the band between 885 and 1090 cm^−1^ provided information about PO_4_^3−^ v1, v3. After baseline correction and normalization, the ratio of the integrated areas of the CO_3_^2−^ v2 contour to the PO_4_^3−^ v1, v3 contour, indicating the C: M value, was measured^37^.

### Microhardness test

The initial SMH of the enamel slides was measured using a Knoop diamond indenter (Duramin-1/-2; Struers, Copenhagen, Denmark) under a 50-gram load for 5 s, according to our previous study^38^,^39^. SMH between 400 and 430 Knoop hardness numbers (KHN) were selected for further nanoscratch resistance testing^40^. After irradiation, five indentations were placed next to the previous measurement at 100-μm intervals. The mean values of all five measurements before and after irradiation were compared.

### Statistical analysis

Data were analysed using Statistical Package for the Social Sciences (SPSS) version 13.0 software (SPSS Inc, Chicago, IL, USA). A paired t-test was used to compare residual depth, residual width, crystallinity, crystal size, C: M ratio and Knoop SMH before and after the irradiation treatments. A *p*-value less than 0.05 (*p < *0.05) was considered statistically significant.

## Conclusions

Based on the present studies, we can conclude that gamma irradiation radiotherapy results in poor wear resistance of enamel. In particular, this alteration may be related to the modification of the crystallography and composition of the enamel induced by irradiation.

## Additional Information

**How to cite this article**: Qing, P. *et al.* Effect of gamma irradiation on the wear behaviour of human tooth enamel. *Sci. Rep.* **5**, 11568; doi: 10.1038/srep11568 (2015).

## Figures and Tables

**Table 1:** Remnant depth and width of scratches on the enamel before and after irradiation under different loads in relation to prism orientation

	**The perpendicular-sectioned enamel slides**			**The parallel-sectioned enamel slides**		
	**Before irradiation (n = 6)**	**After irradiation (n = 6)**	**P value**	**Before irradiation (n = 6)**	**After irradiation (n = 6)**	**P value**
Depth (nm) 20 mN	25 ± 3	29 ± 6	0.34	43 ± 18	52 ± 16	0.45
40 Mn	79 ± 8	101 ± 13	0.03	91 ± 22	154 ± 19	0.005
60 mN	181 ± 10	223 ± 10	0.0009	247 ± 74	381 ± 30	0.02
Width (um) 20 mN	2.8 ± 0.3	2.9 ± 0.2	0.55	3.7 ± 0.4	4.0 ± 0.7	0.36
40 mN	3.5 ± 0.3	3.8 ± 0.2	0.16	4.1 ± 0.5	4.4 ± 0.3	0.27
60 mN	4.7 ± 0.5	5.0 ± 0.3	0.12	4.9 ± 1.4	5.3 ± 0.7	0.56

**Table 2:** The crystallinity, crystal size and C: M value of enamel before and after irradiation in relation to prism orientation

	**The perpendicular-sectioned enamel slides**			**The parallel-sectioned enamel slides**		
	**Before irradiation (n = 6)**	**After irradiation (n = 6)**	**P value**	**Before irradiation (n = 6)**	**After irradiation (n = 6)**	**P value**
Crystallinity (%)	82 ± 5	74 ± 5	0.02	79 ± 8	67 ± 7	0.01
Crystal size (nm)	24 ± 1	27 ± 2	0.02	24 ± 1	27 ± 2	0.02
C:M ratio	0.034 ± 0.007	0.051 ± 0.012	0.01	0.028 ± 0.005	0.033 ± 0.003	0.02

**Table 3:** SMH analysis of enamel before and after irradiation

	**The perpendicular-sectioned enamel slides**			**The parallel-sectioned enamel slides**		
	**Before irradiation (n = 13)**	**After irradiation (n = 13)**	**P value**	**Before irradiation (n = 13)**	**After irradiation (n = 13)**	**P value**
SMH (KHN)	421 ± 28	337 ± 14	p < 0.0001	405 ± 19	328 ± 9	p < 0.0001
